# Effect of Spirulina in Bread Formulated with Wheat Flours of Different Alveograph Strength

**DOI:** 10.3390/foods12203724

**Published:** 2023-10-10

**Authors:** Israel Hernández-López, Cristina Alamprese, Carola Cappa, Virginia Prieto-Santiago, Maribel Abadias, Ingrid Aguiló-Aguayo

**Affiliations:** 1 IRTA, Postharvest Programme, Parc Agrobiotech Lleida, Parc de Gardeny, 25003 Lleida, Spain; 2 Department of Food, Environmental and Nutritional Sciences (DeFENS), Università degli Studi di Milano, 20133 Milan, Italy

**Keywords:** Spirulina, flour strength, bread quality, nutritional properties, sensory acceptability

## Abstract

Consumers within the EU are increasingly asking for natural and healthier food products, which are additive-free and environmentally friendly. The aim of this study was to assess the effects of Spirulina (*Arthrospira* sp.) in bread formulated with four wheat flours with different alveograph strengths. The flours used were Manitoba Flour (00/251), Ground-force wholemeal (Whole/126), Standard Bakery Flour (0/W105), and Organic Bakery Flour (2/W66). Powdered Spirulina biomass was used as a new ingredient with a high nutritional profile and bioactive compounds; incorporation was made at two levels: 1.5% and 2.5% of the flour amount. The same bread recipe was used for all formulations, but for the 1.5 and 2.5% variations, 6 g and 10 g of Spirulina were added, respectively. Antioxidant capacity increased with increasing microalgal biomass. The visual and taste attributes of the breads with microalgae underwent noticeable changes compared to their counterparts without microalgae. Biomass addition significantly (*p* < 0.05) affected bread weight and volume, and different trends were found based on the type of wheat flour. Spirulina-containing breads showed a greener coloration while the microalgae concentration was augmented. The moisture and texture were slightly affected by the addition of the biomass at both levels. The 2.5% concentration samples were well accepted in most cases by consumers, emphasizing the salty flavor as a pleasant feature. No significant sensory differences were observed between samples, and the acceptability index was always higher than 72%. The results show that Spirulina could be an environmentally friendly ingredient for the reformulation of nutritionally enhanced bread with a good texture that is well-accepted by consumers.

## 1. Introduction

Spirulina (*Arthrospira* sp.) is a promising source of nutrients and biologically active compounds as it contains around 50–70% protein, 20% carbohydrates (polysaccharides), 5% lipids, 7% minerals [1], and high levels of phenolic compounds, which, according to some studies, can reduce the risk of cardiovascular diseases (CVD) and cancers, mainly due to their antioxidant properties [2]. As a photosynthetic microorganism, it uses sunlight as an energy source and fixes CO_2_ from the atmosphere, one of the biggest problematic greenhouse gases (GHG) [3]. Other positive environmental impacts of microalgae are their ability to filter water by reducing the levels of phosphates and nitrates [4] and to reach high yields without applying pesticides [5]. Moreover, the use of food processing wastewater has been studied as a feasible way to obtain edible microalgae biomass [6]. In addition, they do not require arable land for cultivation, they have minimal freshwater consumption, and they offer the possibility of being grown in seawater and the potential of replacing non-sustainable soy imports [7].

According to the Spanish Food Composition Database, white bread contains 34.6% moisture, 8.3% protein, 47% carbohydrates, and 1.6% lipids; 100 g of bread has about 240 kcal. It is a product appreciated for its taste, texture properties, convenience, and versatility [8]. It could be a suitable option for Spirulina incorporation since its consumption in Europe is quite high and is generally encouraged as part of a healthy diet. Spirulina could improve the nutritional profile and techno-functional properties of bread for its protein content and biological activity. However, the incorporation of new ingredients into a dough matrix is always challenging. Bread dough can be considered as the dispersion of air or gas bubbles in the matrix created by the gluten and starch present in the flour.

The quality of wheat flours is described by different physico-chemical and rheological properties. According to the Italian Synthetic Index of Quality (ISQ), the parameters on which this Index is based we can find the following:

- “P”, which is also called Dough tenacity, stands for the maximum pressure needed to obtain a dough bubble; its unit is mm H_2_O.
- “L”, which starts measuring at the beginning of the inflation until its rupture; it is measured in mm. This can also be called extensibility.
- “*P/L* Ratio”, which stands for the balance between tenacity and extensibility. A high ratio indicates a resistant dough, while a low ratio indicates a weak and highly extensive dough [9].

The introduction of other proteins in wheat flour dough affects the physical and rheological properties of bread. For instance, Plustea et al. [10] incorporated lupin flour into bread, which resulted in a decrease in bread volume. On the contrary, the incorporation of high protein ingredients can be useful in gluten-free bread where an alternative to the gluten network is necessary to obtain a well-developed structure. For instance, Kahraman et al. [11,12] improved the technological and nutritional quality of gluten-free dough and bread by substituting 25% of the rice flour with chickpea flour. Similarly, in another study, the addition of chickpea flour to wheat flour improved the dough stability during mixing and resulted in a not significantly different bread volume if incorporated at a 10% level [13]. Similar effects might be expected with the introduction of microalgae due to the high protein content. García-Segovia et al. [14] added four different microalgae (*Isochrysis galbana*, *Tetraselmis suecica*, *Scenedesmus almeriensis*, and *Nannochloropsis gaditana*) to bread, and they evaluated the effect on the technological quality, assessing changes in color but not in textural parameters. Lafarga et al. [15] indicated that the introduction of *Tetraselmis* and *Nannochloropsis* microalgae species in bread led to an increase in the total phenolic content and the in vitro antioxidant capacity. Furthermore, a higher content of bio-accessible polyphenols was achieved using these microalgae.

Microalgae biomass is currently marketed as nutraceutical, which includes herbal products, dietary supplements, and isolated nutrients. Further research into Spirulina’s potential therapeutic effects could grow its market as a nutraceutical and generate high revenues [15]. The interest in Spirulina is not only due to its high protein content but also to its easy digestibility as well as its composition of essential amino acids. It is also a source of essential minerals such as Fe, Mn, and Cu. We can also find vitamins such as B, C, and E. Spirulina can also be used to formulate functional products. Biomass can be directly consumed as food, but it can also be incorporated into various food products to improve nutritional quality and act as a therapeutic agent against chronic diseases [16]. However, some characteristics of Spirulina limit its use in food products, such as the green color or the strong salty taste that can be a reason for rejection by consumers. To solve these drawbacks, strategies such as encapsulation are being studied, providing the possibility of better managing these sensory characteristics [17]. However, encapsulation is an expensive process; thus, the direct incorporation of powdered Spirulina into the recipe, at the proper level, is often preferred.

In this context, the aim of this study was the assessment of the effect of Spirulina as an innovative ingredient in the production of bread. This has been assessed by evaluating the rheological, physico-chemical, and nutritional profile of the bread samples, using four different strength flours, enriched with Spirulina. Thus, this study attempts to find the most suitable formulation in terms of not only the nutritional benefit of the consumer but also favoring sustainability and decreasing the environmental impact [18]. 

## 2. Materials and Methods

### 2.1. Wheat Flours and Spirulina Biomass

To assess Spirulina’s effect on bread depending on flour characteristics, wheat (*Triticum aestivum* and *Triticum durum*) flours with different alveograph strength values (W, representing the energy necessary to inflate the dough bubble to the point of rupture) were chosen. Italian flour classification was followed, where 00 is for flours with finer sieving down to whole. According to this, Manitoba Flour is 00, Standard Bakery flour is 0, Organic Bakery flour is 2, and wholemeal flour is whole. The flours were also classified by their strength as follows: Manitoba flour (MF; W = 255 × 10^−4^ J), stone-ground wholemeal flour (WF; W = 126 10^−4^ J), standard bakery flour (BF; W = 105 × 10^−4^ J), and organic bakery flour (OF; W = 66 × 10^−4^ J) [19]. Flours were bought in the online store El Amasadero (Alhaurín El Grande, Málaga, Spain). The microalgae used was organic powdered Spirulina biomass from Ecolife Food (Molina de Segura, Murcia, Spain). The only ingredient of this product is organic powdered Spirulina. It is not genetically modified and is grown and processed without the use of pesticides or synthetic fertilizers.

### 2.2. Mixing Properties of Wheat Flours

Mixing properties of the flours and their Spirulina mixtures (1.5 and 2.5% of flour weight) were assessed by means of a Brabender^®^ Farinograph (Brabender OHG, Duisburg, Germany; 300 g chamber, 30 °C) as reported by Musatti et al. [20]. An amount of 300 g of flour, with the eventual incorporation of Spirulina, was pre-mixed for 1 min in the farinograph mixing bowl. Then, water was added up to reach a dough consistency of 500 ± 20 BU (Brabender Unit) and mixing lasted 25 min. Water absorption (%), and the development time (min) necessary to reach the desired dough consistency were measured, as well as dough stability (min; defined as the time difference between the point where the top of the curve first intersects the 500-BU line and the point where the top of the curve leaves the 500-BU line).

### 2.3. Alveographic Test

The resistance of dough to a three-dimensional extension was measured by means of the Chopin MA 82 Alveograph (Chopin SA, Villeneuve-La-Garenne, France), according to the official standard method (AACC (1983), Approved Methods, 54-30A) [21].

### 2.4. Bread Making Procedure

The amount of flour used for each batch was 400 g since it is the minimum necessary to obtain at least 8 bread loaves. Based on the farinograph water absorption (Section 2.2), the amount of water to be added to each bread formulation was calculated (Table 1). The same quantity of salt (Sal marina fina, Hacendado) and dry yeast (Mauripan, ABMauri, El Prat de Llobregat, Barcelona) was added to all the doughs to obtain the same impact on water absorption. The proportions of these two ingredients were taken from the dough-baking procedure described by Lafarga et al. [22]. Sample codes (Table 1) refer to the flour used (i.e., MF, WF, BF, OF) and the percentage of microalgae added (0, 1.5, and 2.5% of flour).

The ingredients were mixed at room temperature using a bread dough mixer (AM-7000, Orbegozo, Murcia, Spain) equipped with a dough hook at speed 2 and for different mixing times (Table 1) according to the farinographic development time (Table 2) and increased by 1 min in order to ensure complete hydration of the ingredients and a well-developed protein network. After mixing, the dough was placed in an electric oven (SCC WE 101, Rational AG, Landsberg am Lech, Germany) at 30 °C and 80% relative humidity for 15 min for the first leavening stage. Afterwards, the dough was divided into 60 g pieces, molded by hand in 8 tins of 9 × 6 × 4 cm and placed once again in the oven under the same conditions for 45 min for a second leavening step. Afterward, the bread loaves were baked at 200 °C for 20 min in a gas oven (N9PCF/G8001 Serie 900, Fainca HR, Córdoba, Spain). Finally, the loaves were removed from the molds and left to cool at room temperature for 2 h covered with kitchen paper. 

### 2.5. Moisture and Protein Content

The moisture content of flours, Spirulina powder, and bread samples was gravimetrically determined in triplicate according to the AACC Method 44–15.02 and expressed as a percentage. 

The total nitrogen content of flours, Spirulina powder, and bread samples was determined in duplicate by using the Kjeldahl method according to the AOAC Method 950.36. The protein content was then calculated by adopting a conversion factor of 5.7 for flour and bread and 6.25 for Spirulina powder [23].

Moisture and protein content of the mixtures of flour with different amounts of Spirulina were obtained by calculation.

### 2.6. Bread Technological Quality

For each sample, the weight of the eight loaves was measured by using an analytical balance (53R-6KD Gram Precision S.L, Barcelona, Spain). The highest height of each loaf was measured with a digital Vernier caliper (JP Selecta, Barcelona, Spain). The volume of each loaf was calculated using the AACC Method 10–05.01 with some modifications. Briefly, a 1 L plastic beaker was filled with a known amount of quinoa seeds and the volume occupied was registered. Then, the loaf was introduced in the center of the beaker, completely covered by the quinoa seeds, and again the volume was noted. Finally, the bread loaf volume (BV) was calculated with Equation (1).

BV = (Vqb + bread loaf) − Vq(1)

where Vqb is the volume occupied by the known amount of quinoa seeds and the bread loaf; Vq is the volume occupied by the quinoa seeds alone. Considering the loaf weight and BV, the specific volume (mL/g) was calculated.

Bread color was measured using a colorimeter CR-400 Chromameter (Minolta Inc., Tokyo, Japan). CIE values were registered in terms of L* [lightness: black (0)/white (100)], a* [greenness (−60), redness (+60)], and b* [blueness (−60) /yellowness (+60)]. Calibration was carried out using a standard white tile (Y:92.5, x:0.3161, y:0.3321) provided by the manufacturer and the D65 illuminant, which approximates daylight. Three measurements were taken for each loaf in different points (top and sides), while just one for each slice. This represents a total sum of 24 crust color measurements and 8 measurements of crumb color for each formulation.

The Texture Profile Analysis (TPA) was performed as described by Lafarga et al. [24]. A TA.XT2 Texture Analyzer (Stable Micro Systems Ltd., Surrey, UK) connected to Exponent software v.5.0.6.0 was used, equipped with a P/20 aluminum compression probe. Briefly, from each loaf, three slices 10 mm thick were cut; the center of each slice was compressed twice up to 75% deformation at a speed of 2 mm/s, with an interval time of 3 s between one cycle and the other. Results are expressed as hardness (N) and springiness (mm).

Digital pictures of the bread loaves and slices were taken with a digital camera, Digi Eye (VeriVide, Leicestershire, UK), to record the visual aspect (i.e., alveoli crumb structure) of the bread formulations.

### 2.7. Sensory Analysis

Thirty semi-trained panelists, 21 women and 9 men ranging from 18 to 65 years participated in the sensory sessions. These semi-trained panelists were familiar with the evaluated product and had evaluated previous similar products. The sensory evaluation was conducted following the methodology described by Millar et al. [25] with some modifications. Briefly, the evaluation took place in a sensory laboratory with separate booths. The samples were tested within 1 h after baking, preparing 10 mm-thick slices for each formulation. Slices of bread samples obtained with the same flour but different Spirulina concentrations were placed on white polystyrene plates labeled with random codes and presented to the consumers in a randomized order.

Tastings were performed on different days at the same time, with a control sample for each day. A small cup of water was provided to the panelists, so they could drink between sample tastings. The panelists were asked to evaluate the following parameters: texture, taste, and overall acceptability. A 9-point hedonic scale was used, 1 being the worst mark (extremely dislike) and 9 the best one (extremely like). Moreover, the purchase intention (PI) was assessed with a hedonic scale of 5 points where number 1 represented absolutely no intention to purchase and number 5 a clear purchase intention. The acceptability index (AI) was calculated according to Lucas et al. [26]. The Sensorial Sciences and Consumers Committee (CCSC) at the Research and Agrifood Technology Institute (IRTA) authorized (CCSC 3/2021) the experimental procedure of the incorporation of microalgae in bakery products and have considered that it can be developed with guarantees from IRTA-Fruitcentre (PCiTAL. Parc de Gardeny. Edifici Fruitcentre, Lleida, Spain), and, in accordance with the basic legislation in force on Data Protection (Organic Law 3/2018 and General Regulation EU 2016/679) and the legal requirements linked to the ethical principles on Research with human participants (Declaration of Helsinki 1 and The Belmont Report), the measures in place guarantee both data protection and compliance with the ethical principles on research with human participants. 

### 2.8. Protein Digestibility

The determination of in vitro protein digestibility of bread formulations and Spirulina biomass was carried out using the method described by Severini et al. [27] with some modifications. The amount of 0.3 g of sample was diluted in 50 mL of distilled water and adjusted to pH 8.0 with 0.1 M HCl (Sigma Aldrich, St. Louis, MO, USA) and/or 0.1 M NaOH (Sigma Aldrich, St. Louis, MO, USA), while stirring in a 37 °C water bath. A multienzyme solution (Sigma Aldrich, St. Louis, MO, USA) was prepared with 6.25 g of pepsin from porcine gastric mucosa (≥ 400 units/mg protein) and 0.8 g of pancreatin from porcine pancreas (4× United States Pharmacopeia ≥ 100 units/mg protein) in 100 mL of distilled water. The solution was maintained in an ice bath and adjusted to pH 8.0 with 0.1 M HCl and/or 0.1 M NaOH. Afterward, 5 mL of the multienzyme solution was added to the sample suspension with constant stirring at 37 °C. After the addition of the multienzyme solution, the pH of the suspension was recorded with a pH meter (GLP 22, Crison Instruments S.A., Barcelona, Spain) at 15, 30, 45, and 60 s and, after that, every 1 min during the following 14 min. The in vitro digestibility (Y; %) was calculated using the regression equation described by Severini et al. [27] (Equation (2)).

IPVD = 65.66 + 18.10 x ∆ pH_10min_ (2)

where x is the pH of the sample suspension recorded after 15 min digestion with the multienzyme solution.

### 2.9. Total Phenolic Content and Antioxidant Capacity

The total phenolic content (TPC) of the bread samples was determined by the Folin–Ciocalteu method, following the protocol described by Lafarga et al. [28] with some modifications to the extraction process. For the extraction, 3 g of ground sample was added in centrifuge tubes with 15 mL of methanol (Panreac AppliChem, Llinars del Valles, Spain) at 70% kept at 4 °C and mixed for 20 min at room temperature with a Vortex. Afterwards, the sample was centrifuged using a Sigma-3-18 KS centrifuge (Sigma Laborzentrifugen GmbH, Osterode am Harz, Germany) at 12,000 rpm for 20 min at 4 °C and the supernatant was filtered with gauze in a 15 mL Falcon. The Falcon tubes were kept at −80 °C until the Folin–Ciocalteu analysis was performed. The extracts were diluted with methanol at 70% (*v*/*v*) in 2 mL Eppendorf. The dilution was 1:2 (*v*/*v*) for control and 1:10 (*v*/*v*) for the samples containing Spirulina. Absorbance was read at 760 nm using a GENESYS 10S UV-Vis spectrophotometer (Thermo Fisher Scientific, Barcelona, Spain). The TPC was determined in triplicate, and results were expressed as mg of gallic acid equivalents (GAE) per 100 g of dry weight (dw).

To determine the antioxidant activity of breads, the DPPH· scavenging activity assay was performed as previously described by Lafarga et al. [28] on the same extracts prepared for TPC determination. Antioxidant activity performed by DPPH assay was determined in triplicate and expressed as mg of ascorbic acid equivalents (AAE) per 100 g of dw. Samples were read at 515 nm using a GENESYSTM 10S UV-Vis spectrophotometer (Thermo Fisher Scientific, Barcelona, Spain). All the reagents used to determine both the Folin–Ciocalteu and DPPH assays were purchased in (Panreac AppliChem, Llinars del Valles, Spain)

### 2.10. Statistical Analysis

Physico-chemical, rheological, and nutritional results are expressed by mean ± standard deviation (SD) of 3 repetitions. The statistical analysis of the experimental data was completed with the JMP 13 software (SAS Institute Inc., Cary, NC, USA) using a two-way analysis of variance (ANOVA). The comparison of means was made according to the Tukey test with a significance level of 95% (*p* < 0.05). 

## 3. Results and Discussion

### 3.1. Alveograph Test

Table 2 shows the alveograph analysis of flours without microalgae. According to these results, we can see that both the Manitoba and wholemeal flours are the strongest; however, as was said previously, a higher *P/L* ratio is found in resistant and inextensible doughs, meaning that even when the Manitoba has a higher W value, the *P/L* value of the wholemeal is higher. Probably due to the presence of more fiber, which strengthens the flour, as is the case with the organic flour, which presents a higher *P/L* ratio even when it has a lower W value. This is in concordance with the results that Capelli et al. [29] reported. They assessed an alveograph test in flours with different meal contents, and it was shown that the increase in meal in the flour was proportional to the *P/L* ratio increase. They also showed that the water content directly influenced the decrease in dough tenacity (P), while dough extensibility (L) also increased. It was proven that the refining and water content of the analyzed flours have important impacts on the technological properties. With this in mind, according to the needs of every flour and the desired finished product, it is very important to find the optimum level of water, and, of course, the following would be assessing an alveographic analysis of the flours with added microalgae.

### 3.2. Flours and Spirulina Properties

Flour farinographic mixing properties are shown in Table 3 together with the moisture and protein content.

The different flours showed moisture content, ranging from 6.9 to 11.6%. The addition of Spirulina powder (moisture 4.72 ± 0.1%) showed no effect on the moisture content of the flour due to the small amount used. The highest moisture values were found in the MF and BF samples, while OF had the lowest moisture content. The protein content significantly changed according to the type of flour, suggesting that a different protein network can be formed. Generally, the flour strength value is positively correlated with the gluten content and protein quality (i.e., gliadin and glutenin content, which confers unique strength and elasticity properties to the dough). For instance, strong flours, such as Manitoba, usually contain more gluten-forming proteins, which provide elasticity to the dough and a balanced elasticity is related to a higher volume of loaves [30]. This relates well with the obtained results since the protein content was significantly higher for stronger flours. Manitoba (W = 251 × 10^−4^ J) presented the highest protein content (11.7%), followed by the wholemeal flour (W = 126 × 10^−4^ J), while the bakery flour (W = 105 × 10^−4^ J) presented the lowest protein content (7.9%). However, the organic bakery flour (W = 66 × 10^−4^ J), which was the weakest, presented a percentage of protein higher than that of the bakery flour (BF), probably due to the presence of other proteins different from gliadins and glutenins. As expected, the protein content increased proportionally with the addition of Spirulina powder (protein content of 65 ± 1%). 

The mixing properties of the flours allowed the adequate incorporation of water into the dough and the optimal mixing time to be determined. As water absorption was assessed on flour as is, it was expected that flour with the lowest moisture content would have the highest water absorption value. However, the flour water absorption is also related to protein content and quality; in fact, BF, which had the lowest protein content (7.9%), showed a water absorption significantly lower than MF, even if they had similar moisture. Dietary fiber has also been shown to have an important impact on water absorption in baked goods. According to the nutritional label information of these commercial flours, the one with the lowest fiber content is BF (2.9%), while WF and OF were the ones with higher fiber content (9 and 6.9%, respectively). Accordingly, the WF and OF mixtures had the highest water absorption. Water absorption increased with the introduction of Spirulina, probably due to the exopolysaccharides (EPS) present in Spirulina’s external membrane. Similar results were observed by Montevecchi et al. [31], who reported that the addition of Spirulina up to more than 2.5% resulted in sticky doughs, caused by the “hydrocolloid activity” of EPS. However, high water absorption values are desirable in bread baking since they increase yield and decrease bread staling as reported by Monteau et al. [32].

In the studied conditions, MF presented the longest development and stability time. This means it took a long time to reach the given consistency, but it also better withstood the mixing stress; this is typical of strong flours such as Manitoba. The enrichment with Spirulina significantly (*p* < 0.05) reduced the development and stability time by about 25% and 50%, respectively. BF, in contrast, showed a short development time since it only took 2 min to arrive at 500 BU and the stability time was also very short, indicating a weak flour. In this case, the addition of Spirulina significantly increased (*p* < 0.05) the dough stability, probably due to the contribution of protein and fiber. However, in the present work, the leavening phase lasted 60 min, so the use of this flour may not represent a problem. The stability time of WF and OF samples containing Spirulina was significantly (*p* < 0.05) shorter compared to their respective controls, suggesting that Spirulina fiber somehow interfered with the protein network, like in the Manitoba flour [33]. 

In summary, the addition of Spirulina had minimal impact on moisture but increased the protein content of the flour mixtures and raised water absorption. Among the studied flours, the Manitoba flour showed strong characteristics, while adding Spirulina reduced its development and stability times. Bakery flour displayed weaker traits, improved by Spirulina. For the wholemeal and organic flours with Spirulina, stability time decreased, indicating an interaction with the Spirulina fiber. Overall, this investigation offers valuable insights into the nuanced effects of Spirulina on flour properties, presenting potential avenues for its application in the realm of baking as will be discussed in the next sections.

### 3.3. Bread Quality

#### 3.3.1. Technological Properties

Common quality characteristics of the different bread formulations are shown in Table 4. The moisture content of bread samples ranged from 27.4% in BF-1.5 to 37.2% in WF-0. No significant differences were obtained with the incorporation of Spirulina powder, irrespective of the higher amounts of water used in the dough formulations. Moisture values obtained for bread containing Spirulina are comparable to those reported by Garcia-Segovia et al. [14] and Ak et al. [1] for breads containing microalgae.

Product development (i.e., loaf height, volume, and specific volume) is a key quality parameter in bread products. The MF-0 sample had the highest height, volume, and specific volume compared to the other control formulations. This is due to the higher alveographic strength of the Manitoba flour (W = 251 × 10^−4^ J), accounting for a higher resistance to the pressure exerted by gases generated during leavening and for a higher elasticity [19]. The introduction of Spirulina in the Manitoba flour reduced the specific volume of the bread. This can be attributed to the introduction of fiber, which may have weakened the tridimensional structure of the gluten network, while competing with starch for water. This effect was more noticeable in the Manitoba flour due to its capability to form more voluminous breads. Nevertheless, MF-1.5 and MF-2.5 still presented high specific volume and loaf height values, so the microalga introduction would not visually affect bread development. Even though the wholemeal flour was quite strong (W = 126 × 10^−4^ J), its bran content penalized the gluten network of the dough, thus giving an intermediate-to-low volume expansion with respect to the other flours. 

The bakery flour (BF), having low strength and a low protein content, resulted in breads with a low specific volume. Surprisingly, Spirulina addition at 2.5% significantly increased the specific volumes of WF and BF, probably due to an increase in protein. The organic bakery flour (OF) gave intermediately developed bread (i.e., loaf height) characterized by the lowest specific volume and not affected by Spirulina addition at both levels. Achour et al. [34] enriched wheat flour bread with Spirulina biomass at 1 and 3% and observed a slight but significant volume reduction. Other studies also experienced a bread volume reduction after the introduction of microalgae [15,30].

**Figure 1:**
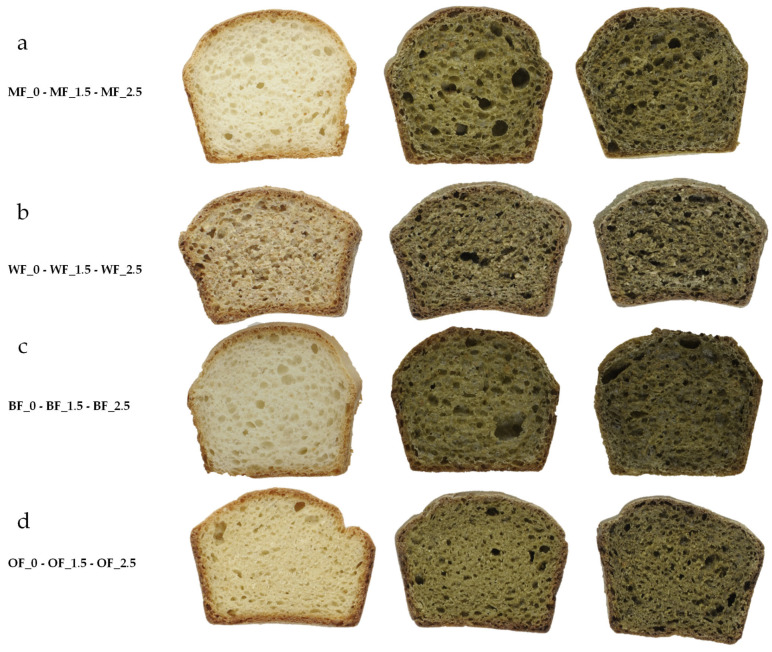
Visual appearance of bread central slice. (**a**) Manitoba flour bread slices in ascending order considering Spirulina content; (**b**) wholemeal flour bread slices in ascending order considering Spirulina content; (**c**) Bakery flour bread slices in ascending order considering Spirulina content; (**d**) Organic flour bread slices in ascending order considering Spirulina content.

Aspects and colorimetric indices of the different bread samples are reported in Figure 1 and Table 5, respectively. A negative relation was observed between the L* values and the concentration of Spirulina. The introduction of microalga also significantly affected a* and b* coordinates, which were lower in all bread formulations with Spirulina, as expected due to the green color of the microalga powder. In fact, crumbs of the different bread formulations visually presented a greenish color, with an intensity increase with higher microalga addition levels. Typically, a TCD > 3 is related to a major color difference and, consequently, visible changes in the appearance of the product are noticed by the consumers [35]. As it is stated, a higher TDC was achieved by OF-2.5 for the crust, while MF-2.5 had the higher crumb TDC.

**Figure 2:**
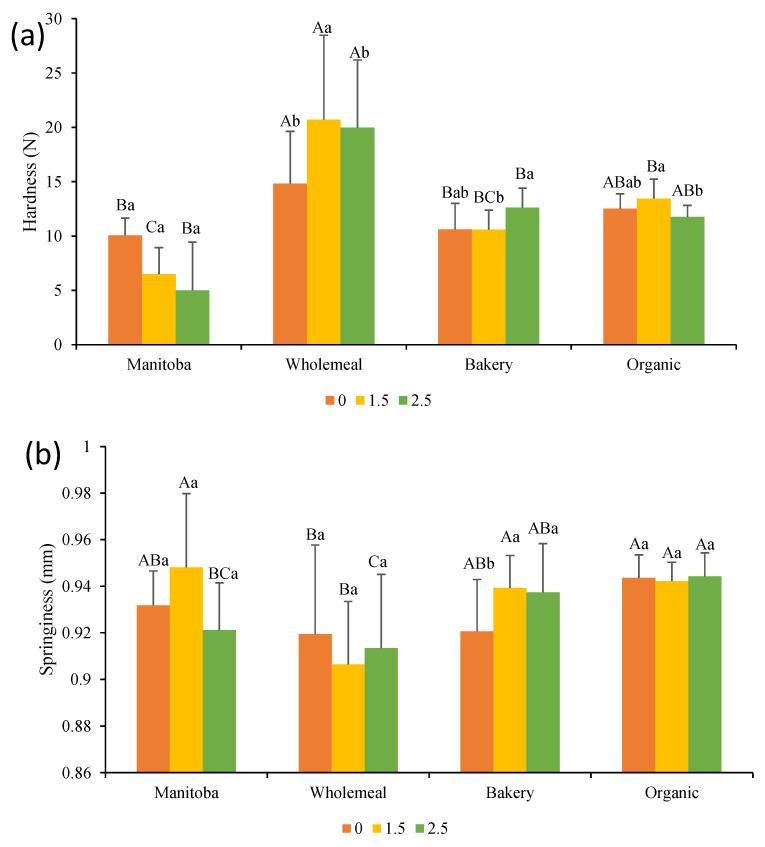
(**a**) Crumb hardness and (**b**) Crumb springiness of bread formulations. Different capital letters indicate significant differences between formulations baked with different flours at the same concentration of Spirulina. Different lowercase letters indicate significant differences between formulations baked with the same flour at a different concentration of Spirulina. The criterion for statistical significance was *p* < 0.05. Numeration: 0 refers to bread without Spirulina addition, 1.5 to incorporation of Spirulina at a concentration of 1.5% in breads, and 2.5 to incorporation of Spirulina at a concentration of 2.5% in breads.

Results of the texture profile analysis (i.e., hardness and springiness) of bread crumbs are shown in Figure 2. The calculated parameters differed among bread formulations containing different flours. In particular, wholemeal flour resulted in harder breads than the BF and MF samples and was characterized by lower springiness, confirming that breads with a higher specific volume are generally softer. In general, the addition of Spirulina did not significantly alter hardness and springiness values, except for WF-1.5, which resulted in a significantly (*p* < 0.05) harder bread than the corresponding control (WF-0). Thus, in line with Garcia-Segovia et al. [14] and Lafarga et al. [15], the addition of microalgae in wheat bread at levels up to 3% did not modify the texture parameters. Figueira et al. [36] enriched rice-flour gluten-free bread with the same microalga and did not observe significant changes in crumb hardness at a concentration of 4%, but an increase of 113% in hardness and a 22% decrease in volume was reported after a 5% level of addition. The volume of breads can be greatly affected by the substitution of flour with microalgae, directly affecting the levels of gluten and natural starches in the flour. With this in mind, it is important to make an accurate selection of microalgae before adding them to the bread mix, as well as an adaptation of the processes to favor mixing and fermentation times. In addition, the amount of water added must be optimized according to the total flour and microalgae mixture.

#### 3.3.2. Sensory Properties

**Figure 3:**
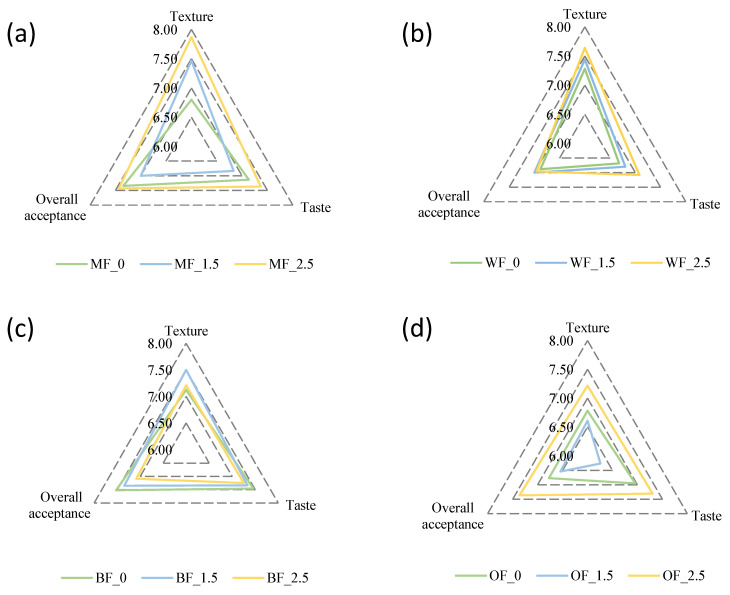
Sensory analysis results for bread formulations baked with (**a**) Manitoba flour, (**b**) wholemeal flour, (**c**) Bakery flour, and (**d**) Organic bakery flour. Sample numeration: MF, Manitoba flour; WF, wholemeal flour; BF, standard bakery flour; OF, organic bakery flour; 0, no Spirulina addition; 1.5, Spirulina incorporation at 1.5% of the flour amount; 2.5, Spirulina incorporation at 2.5% of the flour amount.

No significant differences between control formulations and the ones containing Spirulina were observed for the sensory parameters assessed (Figure 3). According to the panelists, the biggest impact was on the color, since the green color of the bread pieces was not entirely pleasant at first, but even so, when tasted, the overall perception counteracted the first glance. In terms of taste, the panelists indicated that the samples with the 1.5% Spirulina addition were bland, and the 2.5% version was more tasty and pleasant. In terms of texture, both whole wheat and organic flour had the worst perception, possibly because they were less softer samples than bread samples with bread flour or refined flour. In a previous work by Ak et al. [1], the results of the sensory analysis of bread enriched with 10% Spirulina *platensis* were considered satisfactory even if the marine flavor was perceived. No significant differences were observed based on Spirulina incorporation levels or flour strength in the acceptability index (AI, Table 5).

According to Lucas et al. [26], it is necessary to obtain an AI above 70% for a product to be sensory accepted. The AI was higher than 72% in every bread sample. These results agree with a sensory evaluation conducted by Ak et al. [1], where bread containing Spirulina at 2.5% obtained the highest overall acceptability. Purchase intention of the products ranged between 3.4 and 4.2 (assessed using a 5-point hedonic scale) suggesting that the breads would have a good acceptance if commercialized (Table 6).

#### 3.3.3. Nutritional Properties

**Figure 4:**
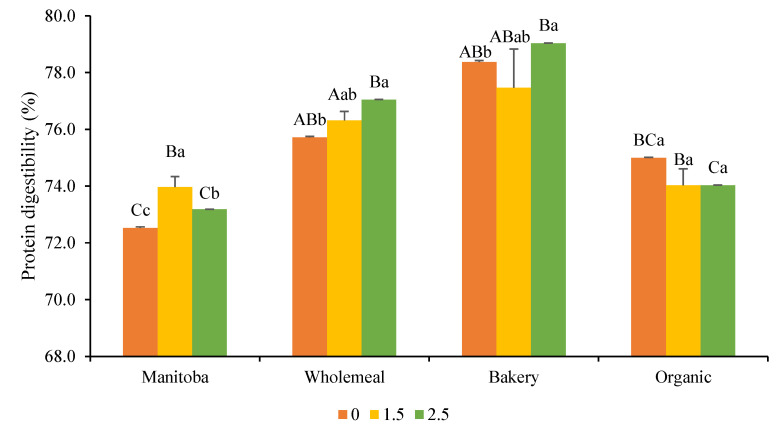
Protein digestibility of bread formulations. Different capital letters indicate significant differences between formulations baked with different flours at the same concentration of Spirulina. Different lowercase letters indicate significant differences between formulations baked with the same flour at a different concentration of Spirulina. The criterion for statistical significance was *p* < 0.05. Numeration: 0 refers to bread formulations without Spirulina, 1.5 to incorporation of Spirulina at a concentration of 1.5% in breads, and 2.5 to incorporation of Spirulina at a concentration of 2.5% in breads.

In this study, the protein digestibility of Spirulina biomass was 78 ± 3%; the high value was due to the lack of cellulose in its cell wall. De Marco et al. [37], after introducing Spirulina in wheat pasta, observed an increase in protein content in the product but a reduction in protein digestibility, probably due to a reaction between proteins and EPS or phenolic compounds interfering with the protein solubilization. However, Batista et al. [38] experienced an increase in protein digestibility in wheat crackers containing 6% of *S. platensis*. In our study, the results seem to be very variable according to the type of flour used (Figure 4). For instance, BF breads presented the highest protein digestibility values, followed by the WF and OF samples, while breads made with MF had significantly (*p* < 0.05) lower digestibility. These results can be explained considering that there are multiple factors that affect protein digestibility in bread, including the characteristics of the flour (such as sifting degree, strength, protein, and fiber content), the tridimensional structure of the dough, kind of gluten, and baking process [39]. Considering the protein digestibility of Spirulina powder, 0.51 g of digestible protein is present in 1 g of Spirulina. In fact, a tendency of Spirulina to increase the protein digestibility in Manitoba and wholemeal flour breads can be observed. For this reason, it could be assumed that higher concentrations of Spirulina would have increased protein digestibility, although this should be better assessed in further studies. According to these results, the potential benefit of consuming bread containing Spirulina could result in an increase in protein consumption just by eating bread, since bread is one of the most consumed staple foods. That is why the use of Spirulina as a protein source has been highlighted for different reasons.

**Figure 5:**
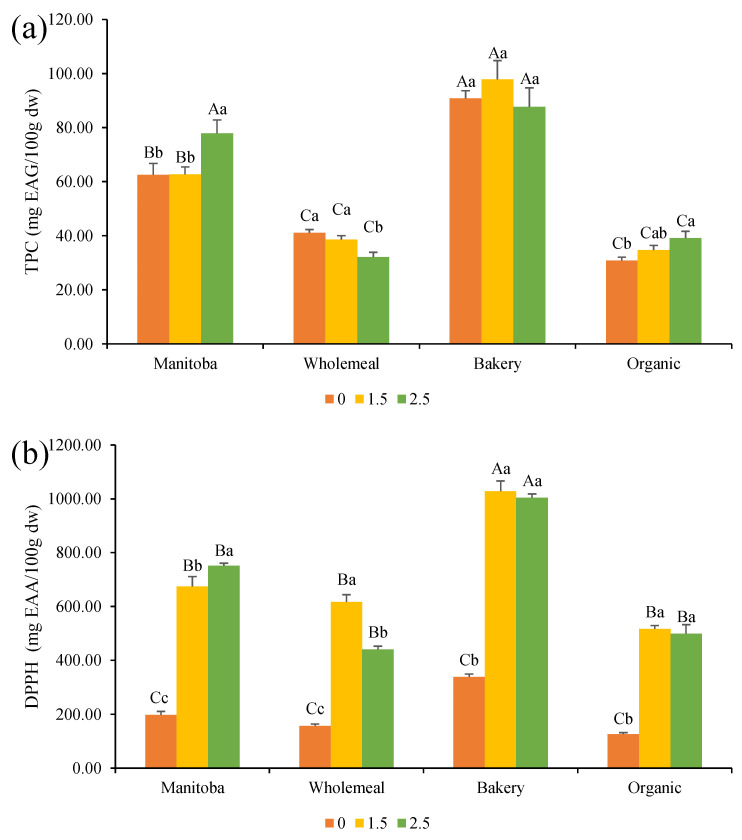
(**a**) Total phenolic content and (**b**) antioxidant activity assessed with the DPPH· scavenging assay of bread formulations. Different capital letters indicate significant differences between formulations baked with different flours at the same concentration of Spirulina. Different lowercase letters indicate significant differences between formulations baked with the same flour at a different concentration of Spirulina. The criterion for statistical significance was *p* < 0.05. Numeration: 0 refers to bread formulations without Spirulina, 1.5 to incorporation of Spirulina at a concentration of 1.5% in breads, and 2.5 to incorporation of Spirulina at a concentration of 2.5% in breads.

The total phenolic content and antioxidant capacity of bread samples are shown in Figure 5. The incorporation of Spirulina at 2.5% significantly increased the TPC in MF and BF breads, due to the high Spirulina TPC, which was equal to 60.2 ± 4.0 mg of GAE/100 g dw. Indeed, microalgae-derived polyphenols are one of the main trends in functional foods for the prevention of cardiovascular diseases and diabetes [40]. Nunes et al. [41] reported that the addition of 1% of fresh *C. vulgaris* resulted in an increase in bread TPC. However, the bread prepared with the commercial *C. vulgaris* powder presented a similar TPC than the control. Rózylo et al. [42] did not observe an increase in the TPC after the addition of brown algae at 2% in gluten-free bread, but a significant increase at a 6% level was observed.

Lafarga et al. [15] evaluated the effect of gastrointestinal digestion performed before determining the TPC and antioxidant activity of breads with microalgae. Results showed that the TPC greatly increased after protein digestion. The authors suggested that it might be due to a longer extraction time and higher bioaccessibility of polyphenols due to the action of digestive enzymes.

Incorporation of Spirulina in bread greatly increased the antioxidant activity measured by the DPPH· assay, possibly due to the presence of microalga polyphenols. However, a previous gastro-intestinal in vitro digestion as the one performed by Lafarga et al. [15] would have provided more accurate results, since Spirulina is rich in phycocyanin, which can interfere with the DPPH· method. Indeed, Lafarga et al. [15] observed an increase in the antioxidant capacity of breads containing microalgae after enzymatic digestion.

The antioxidant capacity of Spirulina was 200 ± 5 µm AAE/100 g dw. As previously reported by Goiris et al. [43], it is due to its phenolic and carotenoid content. Other studies also observed an increase in polyphenols and antioxidant capacity after incorporation of microalgae in pasta [37] or wheat flour tortillas [44], demonstrating the potential of microalgae to increase the antioxidant capacity in foods.

It is denoted that the addition of Spirulina biomass to products such as bread could be a reliable, safe, and rich source of both protein and compounds with specific functions, such as phenolic compounds, inducing antioxidative activity very beneficial for those who consume these products.

## 4. Conclusions

Overall, both the Manitoba flour and traditional baker’s flour had the most favorable results in terms of acceptability and sensory values. In protein digestibility, it was observed that traditional baker’s flour and wholemeal flour had the best performance. In terms of polyphenol content and antioxidant activity, there were similar results between the Manitoba and the wholemeal flour, with the traditional flour being even better. According to the literature reviewed, the results agree with previous studies. This makes it increasingly clear that microalgal biomass is of growing interest as a food ingredient. When it comes to the flour strength, it was shown that the alveograph provides information very valuable to comprehend how a dough could possibly behave. According to the literature reviewed, both wholemeal and organic flours, thanks to their meal content, had a higher *P/L* ratio, being the most resistant flours; furthermore, due to the increase in water absorption facilitated by the microalgae addition, these flours behave similarly to the most refined flours that had lower *P/L* ratios. However, the number of products launched on the market and achieving commercial success is very limited. The most promising food matrices for use as microalgae delivery vehicles, in terms of consumer acceptance, are baked goods, pasta, or condiments. The composition of microalgae and their easy mode of production make them a new and innovative food ingredient for human food staples in the future. On the other hand, Spirulina, due to its high protein content, its desirable essential amino acid profile, and other compounds such as polyphenols, vitamins, and minerals, can be considered as a nutraceutical ingredient. Thus, when added to other food matrices, it provides beneficial health effects such as the prevention of cardiovascular diseases thanks to its high content of antioxidant compounds. In addition, baked goods are particularly relevant, as they are regularly consumed almost everywhere in different presentations, are recognized as staple foods, and are classified as healthy by consumers. Microalgae are rich in sustainable and health-promoting compounds, and their use in food will undoubtedly increase in the coming years. In conclusion, Spirulina shows potential to improve not only traditional breads but also those with different proportions of protein, and it could thus be a nutritional alternative to enrich gluten-free baked goods. As evidenced in this study and in previous studies related to the fortification of foods with microalgae, whether bread or other cereal derivatives, microalgae can be an important source of protein and other bioactive compounds with specific functions in the bodies of consumers. As a source of protein, microalgae are more sustainable than conventional protein sources, such as animal and other vegetable sources, due to their low requirement of soil, water, and other resources compared to conventional farming and agriculture. In addition, by capturing CO_2_ during their respiration process, they contribute to the reduction in CO_2_ in the environment. However, more studies are needed to evaluate the effect of microalgal biomass on the physical, chemical, organoleptic, and bioactive properties of these foods.

## 5. Limitations

Although the present study found interesting results, a limitation should be highlighted. The sample could be considered non-representative of the Spanish population. Therefore, we suggest that future research on the topic should be conducted with larger samples. Sensorially speaking, the visual and taste aspects have the greatest impact on consumers. Among the Western population, the consumption of microalgae is neither widespread nor customary, marking a limitation. The salty taste of microalgae being more or less pleasant depending on who consumes it also defines the percentage of use, decreasing the possibilities for its application in various products and/or recipes.

## Figures and Tables

**Table 1:** Bread formulations and actual dough mixing times.

Sample	Flour (g)	Water (g)	Spirulina (g)	Salt (g)	Yeast (g)	Mixing Time (min)
MF-0	400	246.8	0	8.0	6.0	11.2
MF-1.5	400	252.8	6.0	8.0	6.0	8.9
MF-2.5	400	257.2	10.0	8.0	6.0	8.5
WF-0	400	286.0	0	8.0	6.0	7.3
WF-1.5	400	292.4	6.0	8.0	6.0	7.0
WF-2.5	400	296.0	10.0	8.0	6.0	6.9
BF-0	400	230.4	0	8.0	6.0	3.0
BF-1.5	400	238.0	6.0	8.0	6.0	2.9
BF-2.5	400	240.8	10.0	8.0	6.0	2.9
OF-0	400	306.8	0	8.0	6.0	7.6
OF-1.5	400	313.2	6.0	8.0	6.0	6.9
OF-2.5	400	318.0	10.0	8.0	6.0	7.0

MF, Manitoba flour; WF, wholemeal flour; BF, standard bakery flour; OF, organic bakery flour; 0, no Spirulina addition; 1.5, Spirulina incorporation at 1.5% of the flour amount; 2.5, Spirulina incorporation at 2.5% of the flour amount.

**Table 2:** Alveograph parameters measured by Chopin Alveograph for the different flours.

Sample	P (mmH_2_0)	L (mm)	*P/L* Ratio	W (10^−4^ J)
MF-0	89	68	1.31	251
WF-0	152	17	8.94	126
BF-0	57	51	1.12	105
OF-0	149	9	16.56	66

Sample abbreviations: MF, Manitoba flour; WF, wholemeal flour; BF, standard bakery flour; OF, organic bakery flour; 0, no Spirulina addition.

**Table 3:** Moisture, protein content, and mixing properties (mean ± SD values) of wheat flours and Spirulina mixtures for bread production.

Sample	Moisture (%)	Protein (%)	Water Absorption (%)	Development Time (min)	Stability (min)
MF-0	11.4	11.7 ± 0.2 ^Ab^	61.7 ± 1.8 ^Ca^	10.2 ± 0.9 ^Aa^	21.7 ± 1.8 ^Aa^
MF-1.5	11.3	12.5 ± 0.1 ^Aab^	63.2 ± 2.1 ^Ca^	7.9 ± 0.1 ^Ab^	11.7 ± 0.8 ^Ab^
MF-2.5	11.2	13.2 ± 0.5 ^Aa^	64.3 ± 1.6 ^Ca^	7.5 ± 0.3 ^Ab^	9.8 ± 0.1 ^Ab^
WF-0	9.5	10.3 ± 0.1 ^Ba^	71.5 ± 0.4 ^Bb^	6.4 ± 0.1 ^Ba^	7.0 ± 0.1 ^Ba^
WF-1.5	9.4	10.5 ± 0.2 ^Ba^	73.1 ± 0.1 ^Ba^	6.0 ± 0.1 ^Bb^	3.5 ± 0.1 ^Bb^
WF-2.5	9.4	11.1 ± 0.3 ^Aba^	74.0 ± 0.2 ^Ba^	5.9 ± 0.1 ^Bb^	3.2 ± 0.2 ^Cb^
BF-0	11.6	7.9 ± 0.3 ^Ca^	57.8 ± 0.1 ^Dc^	1.9 ± 0.1 ^Ca^	2.6 ± 0.6 ^Cb^
BF-1.5	11.5	8.5 ± 0.4 ^Ca^	59.5 ± 0.1 ^Db^	1.9 ± 0.1 ^Ca^	4.3 ± 0.1 ^Ba^
BF-2.5	11.4	9.1 ± 0.3 ^Ba^	60.2 ± 0.3 ^Da^	1.9 ± 0.1 ^Ca^	4.5 ± 0.1 ^Ba^
OF-0	6.9	9.8 ± 0.1 ^Ba^	76.7 ± 0.3 ^Ac^	6.6 ± 0.1 ^Ba^	7.0 ± 0.3 ^Ba^
OF-1.5	6.9	9.8 ± 0.2 ^Ba^	78.3 ± 0.1 ^Ab^	5.9 ± 0.2 ^Bb^	5.1 ± 0.3 ^Bb^
OF-2.5	6.9	11.0 ± 0.8 ^Ba^	79.5 ± 0.2 ^Aa^	6.0 ± 0.1 ^Bb^	5.0 ± 0.1 ^Bb^

Different capital letters indicate significant differences between formulations baked with different flours at the same concentration of Spirulina. Different lowercase letters indicate significant differences between formulations baked with the same flour at a different concentration of Spirulina. The criterion for statistical significance was *p* < 0.05. Sample abbreviations: MF, Manitoba flour; WF, wholemeal flour; BF, standard bakery flour; OF, organic bakery flour; 0, no Spirulina addition; 1.5, Spirulina incorporation at 1.5% of the flour amount; 2.5, Spirulina incorporation at 2.5% of the flour amount.

**Table 4:** Technological properties (mean ± SD values) of bread samples made with different flours and Spirulina powder.

Scheme	Moisture (%)	Loaf Weight (g)	Loaf Height (mm)	Specific Volume (mL/g)
MF-0	31.1 ± 1.3 ^Ba^	44.8 ± 0.5 ^Bb^	47 ± 1 ^Aa^	4.01 ± 0.4 ^Aa^
MF-1.5	29.4 ± 0.0 ^ABa^	46.6 ± 0.3 ^Ba^	37 ± 1 ^Bb^	3.14 ± 0.3 ^Ab^
MF-2.5	31.0 ± 0.9 ^Aa^	44.1 ± 0.5 ^Cc^	48 ± 1 ^Aa^	3.71 ± 0.3 ^Aab^
WF-0	37.2 ± 0.9 ^Aab^	44.7 ± 0.5 ^Bb^	42 ± 1 ^Ba^	2.71 ± 0.2 ^Cb^
WF-1.5	31.3 ± 1.3 ^Aa^	44.5 ± 0.4 ^Cb^	39 ± 1 ^Ab^	2.99 ± 0.2 ^Aab^
WF-2.5	34.4 ± 0.2 ^Ab^	41.5 ± 0.6 ^Ba^	41 ± 1 ^Ba^	3.16 ± 0.2 ^Ba^
BF-0	34.6 ± 0.2 ^Aa^	45.8 ± 0.5 ^Ab^	31 ± 1 ^Da^	3.22 ± 0.2 ^Bb^
BF-1.5	27.4 ± 0.2 ^Bc^	47.3 ± 0.6 ^Aa^	30 ± 1 ^Ca^	3.32 ± 0.2 ^Aab^
BF-2.5	32.0 ± 0.4 ^Ab^	45.9 ± 0.3 ^Bb^	31 ± 1 ^Da^	3.60 ± 0.2 ^Aa^
OF-0	28.6 ± 0.5 ^Ba^	38.7 ± 1.1 ^Ab^	38 ± 1 ^Ca^	2.38 ± 0.2 ^Ca^
OF-1.5	28.7 ± 3.0 ^Ba^	38.3 ± 1.2 ^Bb^	38 ± 1 ^ABa^	2.39 ± 0.2 ^Ba^
OF-2.5	32.6 ± 2.4 ^Aa^	37.6 ± 1.0 ^Aa^	37 ± 1 ^Ca^	2.28 ± 0.2 ^Ca^

Different capital letters indicate significant differences between formulations baked with different flours at the same concentration of Spirulina. Different lowercase letters indicate significant differences between formulations baked with the same flour at a different concentration of Spirulina. The criterion for statistical significance was *p* < 0.05. Sample abbreviations: MF, Manitoba flour; WF, wholemeal flour; BF, standard bakery flour; OF, organic bakery flour; 0, no Spirulina addition; 1.5, Spirulina incorporation at 1.5% of the flour amount; 2.5, Spirulina incorporation at 2.5% of the flour amount.

**Table 5:** Color parameters (mean ± SD values) of bread samples made with different flours and Spirulina powder.

Sample	L*		a*		b*		TDC	
	Crust	Crumb	Crust	Crumb	Crust	Crumb	Crust	Crumb
MF-0	52.6 ± 3.9 ^ABa^	63.8 ± 2.2 ^Aa^	15.9 ± 1.1 ^Ba^	2.9 ± 0.1 ^Ca^	28.1 ± 0.7 ^Aa^	11.7 ± 0.7 ^Dc^	-	-
MF-1.5	43.2 ± 3.3 ^Ab^	42.3 ± 2.9 ^Ab^	9.6 ± 1.5 ^Ab^	1.4 ± 0.5 ^Bb^	26.7 ± 1.5 ^Ab^	20.6 ± 0.6 ^ABa^	11.5	23.3
MF-2.5	40.1 ± 2.0 ^Ab^	29.8 ± 1.4 ^Bc^	6.2 ± 0.9 ^ABc^	1.6 ± 0.3 ^Bb^	17.1 ± 1.0 ^Ac^	16.4 ± 0.9 ^Bb^	19.3	34.4
WF-0	51.5 ± 3.5 ^ABa^	64.1 ± 4.1 ^Aa^	16.0 ± 1.1 ^Ba^	2.9 ± 0.1 ^Ca^	26.6 ± 1.7 ^Aa^	13.2 ± 0.8 ^Cc^	-	-
WF-1.5	41.4 ± 1.1 ^Ab^	40.9 ± 2.8 ^Ab^	8.1 ± 0.9 ^Ab^	1.3 ± 0.1 ^Bb^	19.5 ± 0.7 ^ABb^	20.1 ± 0.7 ^ABa^	14.7	24.3
WF-2.5	38.7 ± 2.2 ^ABb^	36.4± 1.6 ^Ac^	15 ± 1.0 ^Ac^	1.5 ± 0.2 ^Bb^	17.6 ± 1.4 ^Ac^	19.1 ± 0.6 ^Ab^	18.1	28.3
BF-0	48.5 ± 1.9 ^Ba^	56.1 ± 2.5 ^Ba^	15.0 ± 1.0 ^Ba^	8.2 ± 0.3 ^Aa^	23.7 ± 1.8 ^Ba^	17.9 ± 0.6 ^Bb^	-	-
BF-1.5	38.4 ± 1.4 ^Bb^	37.8 ± 1.9 ^Bb^	7.8 ± 0.7 ^Ab^	2.4 ± 0.2 ^Ab^	16.4 ± 0.9 ^Bb^	20.1 ± 0.7 ^Ba^	14.4	19.1
BF-2.5	35.4 ± 0.8 ^Cc^	34.8 ± 2.0 ^Ac^	5.4 ± 0.5 ^BCc^	1.5 ± 0.2 ^Ac^	17.8 ± 0.7 ^Bc^	19.9 ± 0.8 ^Aa^	17.1	22.1
OF-0	53.6 ± 2.5 ^Aa^	64.6 ± 0.7 ^Aa^	17.0 ± 2.6 ^Aa^	5.4 ± 0.1 ^Ba^	28.2 ± 2.6 ^Aa^	22.6 ± 0.2 ^ACa^	-	-
OF-1.5	40.9 ± 0.9 ^ABb^	42.7 ± 0.9 ^Ab^	6.2 ± 1.8 ^Ab^	1.1 ± 0.1 ^Ba^	18.3 ± 1.0 ^Cb^	20.7 ± 0.1 ^Ab^	19.4	22.3
OF-2.5	37.0 ± 0.4 ^BCc^	36.3 ± 0.4 ^Ac^	4.8 ± 1.1 ^Cb^	0.9 ± 0.1 ^Bc^	14.4 ± 0.3 ^Bc^	18.9 ± 0.1 ^Ac^	24.8	28.9

Different capital letters indicate significant differences between formulations baked with different flours at the same concentration of Spirulina. Different lowercase letters indicate significant differences between formulations baked with the same flour at a different concentration of Spirulina. The criterion for statistical significance was *p* < 0.05. Sample abbreviations: MF, Manitoba flour; WF, wholemeal flour; BF, standard bakery flour; OF, organic bakery flour; 0, no Spirulina addition; 1.5, Spirulina incorporation at 1.5% of the flour amount; 2.5, Spirulina incorporation at 2.5% of the flour amount.

**Table 6:** Acceptability Index (AI) and Purchase Intention (PI) for bread formulations baked with different flours and Spirulina levels.

Sample	AI (%)	PI
MF-0	81.61 ± 14 ^Aa^	3.9 ± 1.2 ^Aa^
MF-1.5	77.78 ± 15 ^Aa^	3.8 ± 0.7 ^Aa^
MF-2.5	82.59 ± 12 ^Aa^	4.1 ± 0.8 ^Aa^
WF-0	76.44 ± 15 ^Aa^	3.7 ± 1.2 ^Aa^
WF-1.5	77.78 ± 15 ^Aa^	3.5 ± 1.1 ^Aa^
WF-2.5	77.33 ± 15 ^Aa^	3.6 ± 1.2 ^Aa^
BF-0	83.57 ± 12 ^Aa^	4.2 ± 0.8 ^Aa^
BF-1.5	81.64 ± 14 ^Aa^	3.9 ± 1.0 ^Aa^
BF-2.5	78.74 ± 15 ^Aa^	3.8 ± 0.8 ^Aa^
OF-0	75.25 ± 14 ^Aa^	3.4 ± 1.1 ^Aa^
OF-1.5	72.73 ± 15 ^Aa^	3.4 ± 1.2 ^Aa^
OF-2.5	81.82 ± 15 ^Aa^	3.8 ± 1.0 ^Aa^

Different letters indicate significant differences between bread formulations baked with the same flour at a different concentration of Spirulina. The criterion for statistical significance was *p* < 0.05. Sample numeration: MF, Manitoba flour; WF, wholemeal flour; BF, standard bakery flour; OF, organic bakery flour; 0, no Spirulina addition; 1.5, Spirulina incorporation at 1.5% of the flour amount; 2.5, Spirulina incorporation at 2.5% of the flour amount.

## Data Availability

The data used to support the findings of this study can be made available by the corresponding author upon request.
